# Driver Assisted Lane Keeping with Conflict Management Using Robust Sliding Mode Controller

**DOI:** 10.3390/s23010004

**Published:** 2022-12-20

**Authors:** Gabriele Perozzi, Mohamed Radjeb Oudainia, Chouki Sentouh, Jean-Christophe Popieul, Jagat Jyoti Rath

**Affiliations:** 1 LAMIH Laboratory UMR CNRS 8201, Université Polytechnique Hauts-de-France, 59300 Valenciennes, France; 2 Department of Mechanical and Aero-Space Engineering, Institute of Infrastructure Technology Research and Management (IITRAM), Ahmedabad 380026, India

**Keywords:** human-machine shared control, lane keeping assistance, higher order sliding mode, conflict minimization, ADAS, driver assist system

## Abstract

Lane-keeping assistance design for road vehicles is a multi-objective design problem that needs to simultaneously maintain lane tracking, ensure driver comfort, provide vehicle stability, and minimize conflict between the driver and the autonomous controller. In this work, a cooperative control strategy is proposed for lane-keeping keeping by integrating driving monitoring, variable level of assistance allocation, and human-in-the-loop control. In the first stage, a time-varying physical driver loading pattern is identified based on a relationship between lateral acceleration, road curvature, and the measured maximum driver torque. Together with the monitored driver state that indicates driver mental loading, an adaptive driver activity function is then formulated that replicates the levels of assistance required for the driver in the next stage. To smoothly transition authority between various modes (from manual to autonomous and vice versa) based on the generated levels of assistance, a novel higher-order sliding mode controller is proposed and closed-loop stability is established. Further, a novel sharing parameter (which is proportional to the torques coming from the driver and from the autonomous controller) is used to minimize the conflict. Experimental results on the SHERPA high-fidelity vehicle simulator show the real-time implementation feasibility. Extensive experimental results provided on the Satory test track show improvement in cooperative driving quality by 9.4%, reduction in steering workload by 86.13%, and reduced conflict by 65.38% when compared with the existing design (no sharing parameter). These results on the cooperative performance highlight the significance of the proposed controller for various road transportation challenges.

## 1. Introduction

Advanced driver assist systems (ADASs; acronyms of this manuscript are defined in the Acronyms section) such as lane keeping assist (LKA), adaptive cruise control (ACC), and collision avoidance (CA) systems have been widely employed in commercial vehicles. These systems greatly reduce the workload of human drivers and reduce the risk of accidents, and crashes by warning or supporting the driver for particular maneuvers [1]. The ADASs developed for semi-autonomous driving scenarios can be categorized into human-guided, human-supervised, and human-assisted architectures [2]. In recent works, it has been established that driver-in-the-loop (DiL) human-assisted ADAS architectures can be employed to address various human–machine interaction (HMI) challenges inclusive of authority allocation [3], the transition of authority [4], conflict management [5], and human driver workload reduction and skill enhancement [6]. Such cooperative driving architectures have been explored for adaptive cruise control, collision avoidance systems, and lane departure/keeping systems among others [7,8]. To design cooperative control architectures for ADAS, DiL architectures are typically formulated by integrating driver attributes such as workload, experience, and skill in the control design. For effective action which reflects such attributes, various driver models based on neuromuscular dynamics [9], data-driven [10], hand impedance [11], and vision/preview have been developed [5]. In this work, the avenue of cooperative control for lane-keeping assistance (LKA) systems considering the steering input (angle or torque) as a control signal is explored with a focus on HMI management and vehicle positioning error minimization.

### 1.1. State of the Art

Many works can be found in the literature dealing with the design of controllers for trajectory following [12]. Among of all the robust controllers, the sliding mode law is worldwide recognized as one of the most effective to reject external matched perturbations [13], so they can be used to reject perturbations that affect road vehicles. The system disturbances and parameter uncertainties introduced by human–machine cooperation driving are also inevitable. Ref. [14] proposed a control method to solve the above problems. Optimization algorithms have also been used to reduce the computational cost of implementing the control law in real-time applications [15]. Active fault-tolerant controllers have been largely used to increase plant availability and reduce the risk of safety hazards, preventing simple faults from developing into serious failure [16,17]. The last decade witnessed a great development of automated driving vehicles and vehicle intelligence. The significant increment of machine intelligence poses a new challenge to the community, which is the collaboration between human drivers and vehicle autonomy. In [18], a literature review was conducted and perspectives on the human behaviors and cognition (HBC) for ADVs toward human-autonomy (H-A) collaboration were proposed.

Various cooperative control architectures have been proposed in [5,7,8,19,20] based on DiL designs. In [21], a driver model using a weighting process of visual guidance from the road ahead and haptic guidance from a steering system for a lane-following task were proposed. In [3,22,23,24], haptic feedback from the steering wheel was used to ensure both driver and the autonomous controller participated in the driving action. In [25], an extended shared steering control system with an authority adaptive allocation model was proposed to improve the reliability of the shared steering control system, and weaken the influence of uncertain driver behavior on driving safety. Ref. [26] presented a shared control framework based on handling inverse dynamics and driving intention for lane changing, in particular, the influence of the driver’s lane-changing start point and end point is considered in the design of the shared controller. In [6], a cooperative control approach for lane keeping based on $H_{2}$ preview control was proposed by incorporating a neuro-muscular driver model. Similarly, in [20], a haptic shared control between driver and e-copilot considered the use of driver torque as haptic feedback to design T-S fuzzy controllers for lane keeping. In [19], for varying driver steering characteristics such as delays, and preview time, a DiL gain-scheduling $H_{\infty }$ robust shared controller was proposed. These approaches typically validated the cooperative performance of the DiL design for lane-keeping tasks in presence of driver parameter uncertainty and environmental disturbances such as crosswinds, and road curvature. Although efficient lane-keeping performance under various driving conditions was validated, issues of conflict between human driver and autonomous controller, driver workload management and performance enhancement were not explicitly addressed.

Driver workload typically characterizes the driving action required by the human driver to perform a typical task. Based on monitored cognitive states (mental workload) and physical driving effort (physical workload) applied by the driver, the workload can be categorized into under-load, normal and over-load regions [5,27]. The mental workload of the driver reflects the state of involvement of the driver in the driving task. Typically, driver state of drowsiness [5,28], the intention of driving action [28], and meticulous steering action [29] are employed as indicators of the mental workload. The physical workload of the driver can be determined by monitoring the driver torque/steer input applied, and the steer reversal rate. The objective of a cooperative LKA strategy is then adapting the driver activity in terms of workload into the controller design for effective management of HMI and keeping vehicles on the lane. In [30], an optimal modulation policy was designed with a cost function, then a nonlinear stochastic model predictive approach was used to solve the cost function subjected to probabilistic uncertainties in human’s biomechanics. In [27], the relationship between driver workload and level of assistance required was explored for the design of an LKA controller to improve driver performance. Takagi-Sugeno (T-S) models [31,32] used driver activity functions considering driver state, torque, and intention, which replicate the level of assistance required during a typical task [27].

The conflict between the human driver and the autonomous controller typically occurs when both agents have different actions for the same driving task. Such scenarios arise during the transition of authority between the agents, sudden maneuvers executed by driver/automation which is not predicted by the other agent, and during extreme maneuvers i.e., sharp curve negotiation. In [4,6], based on cooperative status detection, a conflict-free smooth transition of authority between human driver and autonomous controller was proposed. Similarly, in [23], conflict mitigation by adapting the parameters of the controller with respect to individual drivers was proposed. Extending the work of [31], a cooperative control approach employing T-S models was proposed in [5] to perform lane keeping and conflict minimization simultaneously. In [33], a haptic control architecture was developed for the smooth transition of control authority with adaptation to driver cognitive workload. In the works of [6,19,20,31,32], the controllers designed were based on the linear bicycle model which did not account for varying tire friction forces. The works in [6,19] assumed constant longitudinal speed in the design of lane-keeping controllers. Further, conflicts between the driver and the automated driving system were not explicitly addressed in [19,32]. In [5,31,33], by the design of shared control dependent on driver attributes, the issue of conflict between the driver and automated system was addressed for variable longitudinal speeds and fixed longitudinal speeds. However, these works were analyzed for the linear bicycle model that did not consider the aspect of saturated tire friction forces during extreme maneuvers.

### 1.2. Proposed Methodology

To account for tire-force non-linearities and environmental disturbances, management of HMI between human drivers and autonomous controller with respect to driver workload, and conflict management, a robust cooperative control approach is proposed in this work. Based on the non-linear representation of tire-friction dynamics [34] integrated with a human driver model developed using visual cues [5], a DiL design is formulated. The HMI between the human driver and the driver torque is then developed based on adaptation to driver workload and subsequent driver performance. For adaptation to driver performance, a non-linear representation of driver activity based on physical and cognitive workload is formulated. For quantifying adaptive physical workload, a rule-based logic is used to explore the relationship between lateral acceleration, predicted road curvature, and maximum driver torque. Based on the developed DiL model dynamics, a novel robust nonlinear feedback controller based on adaptive higher order sliding mode (HOSM) [35,36] is developed for the system. The conflict is managed by the introduction of a sharing parameter, which is a function for driver and assistance torques in the input-dependent sliding surface. The developed feedback control is then modulated using the non-linear function developed on the relationship of driver performance-level of assistance required, for effective HMI management. The closed-loop stability of the time-varying system dynamics involving the non-linear modulating function, DiL dynamics, and environmental disturbances is then established.

### 1.3. Contribution

The main contributions of this work are:

- The introduction of a shared control parameter into the control design to minimize conflict between the human driver and automated driving system.
- The design of a novel higher-order sliding mode control algorithm with linear and nonlinear terms.
- The addressing of multiple objectives of lane position error reduction, enhancement of driver satisfaction, and conflict management.

The manuscript is organized as follows. Section 2 introduces the driver–vehicle–lane model. Section 3 focuses on the design of the proposed controller. Extensive discussions about the performance of the proposed approach with regard to lane position error reduction, driver satisfaction, and the influence of the conflict parameter are provided in Section 4.

## 2. Problem Formulation: Driver Adapted Lane Keeping

The time-varying dynamics governing a DiL vehicle model in the presence of environmental disturbances for lateral control and the problem of designing a closed-loop controller to manage the HMI between a driver and an autonomous controller are discussed in this section. The symbols of this manuscript are defined in the Nomenclature section.

### 2.1. DiL Modeling: Vehicle-Road-Driver Dynamics

The DiL model development is carried out by integrating the vehicle’s lateral and yaw motion dynamics with the steering column dynamics, the lane tracking dynamics, and a linear model of the human driver’s torque. The governing dynamics for the lateral motion of the vehicle under assumptions of negligible influence of the longitudinal dynamics can be efficiently represented using the non-linear bicycle dynamic model [1,37] as in Equation (2).

(1) $$\begin{array}{cc} Mv_{x}\dot{\beta } & =F_{yr}+F_{yf}cos\left(\delta_{f}\right)-Mv_{x}{\dot{\psi }}_{v}+F_{w} \end{array}$$

(2) $$\begin{array}{cc} I_{z}{\ddot{\psi }}_{v} & =l_{f}F_{yf}cos\left(\delta_{f}\right)-l_{r}F_{yr}+M_{w} \end{array}$$

where β is the side slip angle, δ is the steering angle, ψ is the heading angle, $F_{yf},F_{yr}$ are the front and rear friction forces, $F_{w}$ is the crosswinds force, and $v_{x}$ is the longitudinal velocity. To represent the tire–road friction conditions, several linear, adaptive, uncertain, and nonlinear models like the Brush-Tire (BT) friction model, LuGre friction model among others are employed [38]. Although the nonlinear models represent the dynamic characteristics of tire–road friction, these models are not easily applicable in control approaches due to their highly complex behavior and dynamics. The linear uncertain friction model [39] has been employed in this work for controller development. The lateral tire friction forces and the self-align torque of the steering wheel are then given as in Equations (3) and (4).

(3) $$\begin{array}{cc} F_{yi} & =2C_{pi}\alpha_{i}+\Delta F_{i} \end{array}$$

(4) $$\begin{array}{cc} T_{s} & =\frac{K_{p}t_{p}F_{yf}}{R_{s}} \end{array}$$

with $\alpha_{f},\alpha_{r}$ denoting the front and rear slip angles, $T_{s}$ denoting the self-aligning torque, and $\Delta F_{i}$ denoting the lumped uncertain part of the tire friction forces indicative of the effects of changing road conditions, tire pressure variations, saturation, etc., which can be modeled using any of the above-discussed dynamic friction models. The variable $K_{p}\in \left(0,1\right]$ is a ratio denoting the level of assistance from the active steering system. In the absence of any active steering support, the value of $K_{p}=1$. The front and rear slip angles under small angle assumptions are given as in Equation (6).

(5) $$\begin{array}{cc} \alpha_{f} & =\delta_{f}-\frac{\beta v_{x}+l_{f}\dot{\psi_{v}}}{v_{x}} \end{array}$$

(6) $$\begin{array}{cc} \alpha_{r} & =\frac{\beta v_{x}-l_{r}\dot{\psi_{v}}}{v_{x}} \end{array}$$

Under the small angle assumptions, the above non-linear bicycle model dynamics appropriately represent the vehicle motion for low curvature roads and have been widely employed for shared lateral control [5,7].

**Figure 1:**
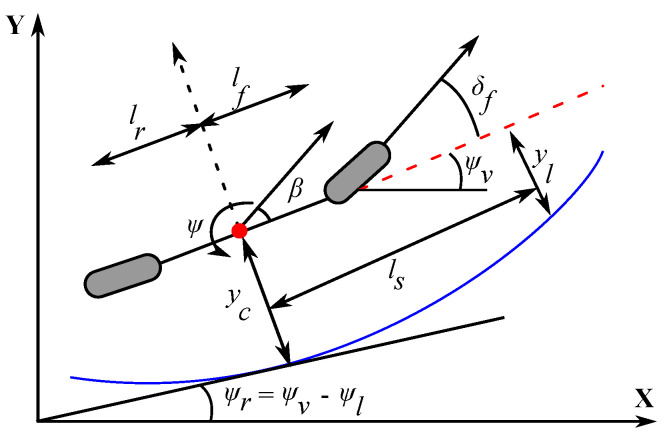
The nonlinear bicycle model of vehicle.

The vehicle’s lane tracking performance can be modeled using two error variables, $y_{l}$ and $\Psi_{l}$, which indicate the lateral deviation error and the orientation error of the vehicle with respect to the lane center-line at a specified look-ahead distance as shown in Figure 1.

These lane errors are readily obtained using vision-based sensors from the vehicle perception unit. The dynamics of these error variables are given, as [5], in Equation (8).

(7) $$\begin{array}{cc} {\dot{y}}_{l} & =\beta v_{x}+l_{s}\dot{\psi_{v}}+\Psi_{l}v_{x}; \end{array}$$

(8) $$\begin{array}{cc} {\dot{\Psi }}_{l} & =\dot{\psi_{v}}-\rho_{r}v_{x} \end{array}$$

with $y_{l},\Psi_{l}$ representing the lateral offset error and the heading error respectively. With the road-vehicle dynamics considered, the interaction between the human driver and the vehicle is then modeled by considering the steering-column dynamics with only basic assist provided [5,7] as in Equation (9).

(9) $$I_{s}{\ddot{\delta }}_{d}=T_{d}+T_{a}-T_{s}-B_{u}{\dot{\delta }}_{d}$$

where $T_{d},T_{a}$ represent the driver and the automation torques, respectively. Integrating the dynamics (8) and (9), an autonomous controller can be designed to generate the assistance torque $T_{a}$ which can maintain the vehicle on the lane. Further, the consideration of the steering column dynamics also helps in informing the human driver of the external road conditions directly.

### 2.2. HMI Management: Driver Workload-Level of Assistance

**Figure 2:**
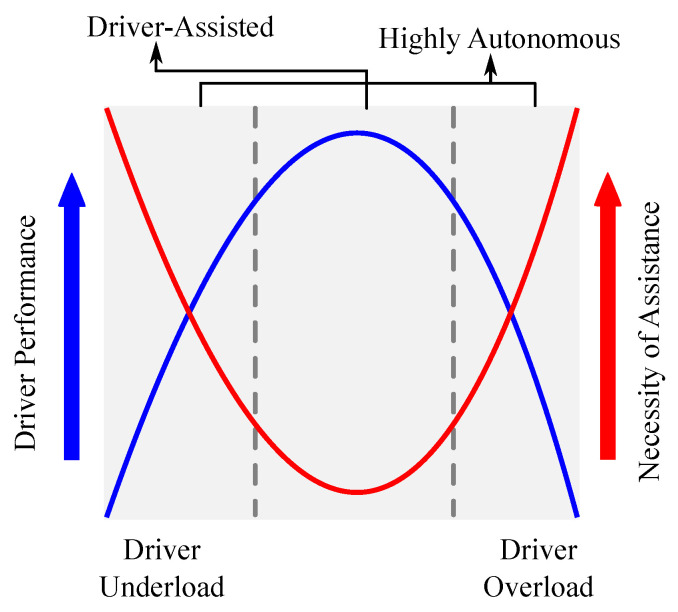
The driver workload and corresponding level of assistance required.

Driver-adaptive LKA systems intend to provide assistance to human drivers for difficult and adverse scenarios [5,40,41,42]. Specifically, adaptation techniques are designed such the physical and mental workload of drivers during driving can be easily managed. Using measured vehicle responses such as steering torque, steering wheel reversal rate, and jerk among others, the physical workload of a driver is quantified [5,27,42]. Similarly, based on measured driver responses such as gaze monitoring, drowsiness, and intention to perform a maneuver, the mental workload of a driver can be quantified [5,7,9]. Integrating both these indicators via a nonlinear mapping and relating them to driver performance, various adaptive functions have been proposed by our research group for shared lane-keeping tasks [5,28,42]. On similar lines, we consider the use of normalized driver torque and driver distraction levels as indicators of the driver’s physical and cognitive workloads, respectively. The entire procedure is carried out in three steps as shown below:

- *Identification of driver workload*: The measured driver torque at the steering wheel is typically dependent on many factors such as road curvature, lateral acceleration, the preview time, and the far point distance, and dynamics of the human arm among others. In this work, the adaptive driver torque $T_{dm}$ for various drivers/driving scenarios is computed using a simple rule-based logic with the inputs being lateral acceleration and predicted road curvature [43]. With the increase in lateral acceleration and road curvature, the value of the $T_{dm}$ increases, to show more physical workload of the driver. Mathematically, the normalized maximum driver torque is represented in Equation (10).
  (10) $$T_{dn}=\left|T_{d}/T_{dm}\right|$$
  Similarly, the mental workload is accounted for by the driver state (DS∈[0,1]) which categorizes the driver’s involvement into different levels such as attentive, sleepy, drowsy, and distracted. With the increase in DS the driver is more involved in the driving task and vice-versa. In the case when DS=0, the driver is completely distracted, and when DS=1, the driver is actively involved in the driving task. For practical purposes, the DS is obtained from the driver monitoring unit (DMU) installed in vehicles comprising of a vision system to monitor driver activity [44]. It is of note that, although different states of driver are monitored, generally the output of the DMU is binary indicating an active driver or a distracted driver [28].
- *Mapping driver workload to activity*: In the context of driver workload, effective driver performance decreases with an increase in workload levels. Similarly, for low activity (corresponding low workload) level, also the performance of the driver is low, as the driver is not significantly involved in the driving task. Analytically, this relationship is expressed as in Equation (11).
  (11) $$\gamma =1-e^{{\left(\sigma_{1}T_{dN}\right)}^{\sigma_{2}}DS^{\sigma_{3}}}$$
  where γ∈[0,1] indicates driver activity, $\sigma_{1}=2$, $\sigma_{2}=3$, and $\sigma_{3}=3$ selected appropriately to consider the degree of influence of the physical and cognitive components on the driver activity. This relationship is presented graphically in form of a U-shaped function in [27].
- *Activity-based level of assistance generation*: The level of assistance (LOA) required to complete a driving task can be determined similarly to [27], by using the inverse-U relationship between driver performance and LOA. Considering the objective of providing high assistance to the driver during under-load and over-load (i.e., low activity) regions, an analytical mapping for driver performance-LOA is defined as in Equation (12).
  (12) $$\mu \left(\gamma \right)=\frac{1}{1+|\frac{\gamma -p_{3}}{p_{1}}{|}^{2p_{2}}}+\mu_{min}$$
  The time-varying parameter $\mu \left(\gamma \right)\in \left[\mu_{min},1\right]$ represents a modulation factor that relates the driver workload-based performance with the LOA for task completion. The parameters $p_{1}=0.355$, $p_{2}=-2$, $p_{3}=0.5$ are chosen to replicate the U-shaped relationship as discussed in [27] and shown in Figure 2. A minimum assistance level of $\mu_{min}=0.2$ is used to consider the influence of sensor noise, drift, etc.

The computed level of assistance function can be then used to modulate the assistance torque $T_{a}$ and thus adapt the autonomous control action to the driver as in Equation (13).

(13) $$T_{a}=\mu \left(\gamma \right)T_{fb}$$

where $T_{fb}$ is a robust feedback control torque to be designed. Employing the modulated assistance torque, the HMI between the driver and the autonomous controller can be effectively managed for completing a specific driving task.

## 3. Robust DiL Lane Keeping Control: A HOSM Approach

The shared control between the human driver and an LKA controller typically focuses on tracking the desired reference while improving the driver comfort [7,20,42].

### 3.1. Control Oriented DiL Modeling

For DiL tasks, we incorporate the influence of driver effort by using a two-point visual driver torque model [5] for developing the control specific model as in Equation (14).

(14) $$T_{d}=K_{c}\theta_{near}+K_{a}\theta_{far}$$

with $\theta_{near},\theta_{far}$ representing the near and far visual points of the driver along a road curvature. Based on information of these angles, the driver generates anticipatory action and compensatory action corresponding to the near and far angles respectively. Subsequently, he/she predicts the future road and generates the anticipated steering action before entering the curve based on the far visual angle. The compensatory behavior of the driver is emphasized for lane-keeping aspects. This driver behavior is represented using the anticipatory and compensatory gains $K_{a}$ and $K_{c}$ respectively as shown in (14). For further details, please refer to [5].

Integrating the above dynamics in Equations (2) and (14), a DiL lane-keeping model of the following form can be formulated in Equation (15).

(15) $$\dot{x}\left(t\right)=A\left(t\right)x\left(t\right)+B\left(t\right)T_{a}\left(t\right)+E\left(t\right)\omega \left(t\right)$$

with the states as $x={\left[\begin{array}{cccccc} x_{1} & x_{2} & x_{3} & x_{4} & x_{5} & x_{6} \end{array}\right]}^{T}={\left[\begin{array}{cccccc} \beta & \dot{\psi_{v}} & y_{l} & \Psi_{l} & \delta_{d} & \dot{\delta_{d}} \end{array}\right]}^{T}$. The system matrices are given as in matrices (17).

(16) $$\begin{array}{cc} A(t) & =\left[\begin{array}{cccccc} a_{11} & a_{12} & 0 & 0 & a_{15} & 0\\ a_{21} & a_{22} & 0 & 0 & a_{25} & 0\\ 0 & 1 & 0 & 0 & 0 & 0\\ v_{x} & l_{s} & v_{x} & 0 & 0 & 0\\ 0 & 0 & 0 & 0 & 0 & 1\\ a_{61} & a_{62} & a_{63} & a_{64} & a_{65} & a_{66} \end{array}\right],B=\left[\begin{array}{c} 0\\ 0\\ 0\\ 0\\ 0\\ b_{1} \end{array}\right] \end{array}$$

(17) $$\begin{array}{cc} E(t) & ={\left[\begin{array}{cccccc} e_{1} & e_{2} & 0 & 0 & 0 & 0\\ 0 & 0 & 0 & -v_{x} & 0 & 0\\ e_{1} & e_{2} & 0 & 0 & 0 & e_{3}\\ e_{4} & e_{5} & 0 & 0 & 0 & 0 \end{array}\right]}^{T},w\left(t\right)={\left[\begin{array}{c} F_{w}\\ \rho_{r}\\ \Delta F_{yf}\\ \Delta F_{yr} \end{array}\right]}^{T} \end{array}$$

with $a_{11}=-2\left(C_{f}+C_{r}\right)/Mv_{x}$, $a_{12}=2\left(l_{r}C_{r}-l_{f}C_{f}\right)/Mv_{x}^{2}$, $a_{15}=2C_{f}/Mv_{x}R_{s}$, $a_{21}=2\left(l_{r}C_{r}-l_{f}C_{f}\right)/I_{z}$, $a_{22}=-2\left(l_{r}^{2}C_{r}+l_{f}^{2}C_{f}\right)/I_{z}v_{x}$, $a_{25}=2C_{f}l_{f}/I_{z}R_{s}$, $a_{61}=\left(2C_{f}\eta_{t}/\left(I_{s}R_{s}\right)\right)+K_{c}\tau_{a}^{2}a_{21}$, $a_{62}=\left(2C_{f}l_{f}\eta_{t}/\left(I_{s}R_{s}v_{x}\right)\right)+K_{c}\left(\tau_{a}+\tau_{a}^{2}a_{22}\right)$, $a_{63}=K_{a}/I_{s}$, $a_{64}=K_{a}/\left(I_{s}v_{x}\tau_{p}\right)$, $a_{65}=\left(-2C_{f}\eta_{t}/\left(I_{s}R_{s}^{2}\right)\right)+K_{c}\tau_{a}^{2}a_{25}$, $a_{66}=-B_{s}/I_{s}$, $b_{1}=1/I_{s}$, $e_{1}=1/Mv_{x}$, $e_{2}=l_{w}/I_{z}$, $e_{3}=l_{f}/I_{z}$, $e_{4}=-l_{r}/I_{z}$, and $e_{5}=-K_{p}\eta_{t}/I_{s}R_{s}$.

The autonomous assistance torque $T_{a}$ for completing the driving task in the presence of disturbances ω and the uncertainties Δ can be now designed. Integrating the assistance modulation factor developed earlier, the DiL model used for controller design can be expressed as in Equation (18).

(18) $$\dot{x}=A\left(t\right)x\left(t\right)+B_{1}\left(t\right)T_{fb}\left(t\right)+E\left(t\right)\omega \left(t\right)$$

with $B_{1}=B\mu \left(\gamma \right)$ and $T_{fb}$ as the control torque to be designed for stabilizing the DiL system.

### 3.2. Control Objectives for LKA

The control objectives for the above DiL lane-keeping task are formulated as:

- Minimization of lane tracking errors: The lane tracking errors as given in Equation (8) comprise the errors lateral deviation and the heading angle. To quantify the lane error at a look-ahead distance, the parameter $e_{l}$ is defined as in Equation (19).
  (19) $$e_{l}=y_{l}+l_{s}\Psi_{l}$$
  The control objective is then to ensure that the front wheels of the vehicle are simultaneously located in strip (±d=1.5 m) along the lane center line. In other words, the following condition in Equation (20).
  (20) $$|e_{l}|\leq \frac{2d-w_{r}}{2}$$
  where $w_{r}$ denotes the width of the vehicle.
- Improvement of driver comfort: The comfort of the driver while navigating the road can be understood as a measure of the vibrations or oscillations at the steering wheel. As such, the steering rate ${\dot{\delta }}_{d}$ or the lateral acceleration can be used as a measure to quantify the driver comfort [45].
- Conflict Minimization: The mismatch of control actions between the human driver and the autonomous controller categorized as conflict, must be minimized for having a good shared control performance [5]. This can be achieved by passing over the authority to the human driver. Accordingly, the following fictional state is introduced to achieve the above action in Equation (21).
  (21) $${\dot{x}}_{cf}=T_{d}^{s}-\lambda_{c}T_{a}$$
  where $\lambda_{c}$ is any positive parameter reflecting the level of sharing, and $T_{d}^{s}$ represents the driver torque measured at the steering wheel. In case of conflict, the value of ${\dot{x}}_{cf}$⟶0. In such a case, it can be deduced that $\lambda_{c}T_{a}\approx T_{d}^{s}$. Hence, by the appropriate design of the parameter $\lambda_{c}$, the influence of the assistance torque can be reduced.

For the above control objectives, we now propose a robust HOSM controller for the DiL dynamics in Equation (18) to design the torque $T_{fb}$ and $T_{a}$ subsequently.

### 3.3. Robust HOSM Controller

Integrating the above control objectives, a linear error surface to be regulated can be defined as in Equation (22).

(22) $$\sigma_{c}=k_{1}e_{l}+k_{2}{\dot{e}}_{l}+k_{3}{\dot{\delta }}_{d}+k_{4}x_{cf}$$

for the gains $k_{i}>0$, i=1…4 designed to ensure convergence of the error surface. To stabilize the DiL system and ensure that the tracking error $\sigma_{c}$ converges to a stable equilibrium, the following finite time controller is proposed.

**Theorem 1.**  *For the DiL system in Equation (18), the feedback control $T_{fb}$ which ensures that the tracking error $\sigma_{c}$ in Equation (22) converges to a practically stable equilibrium can be designed as in Equation (23).*

(23) $$T_{fb}=\frac{1}{\Omega_{u}\mu \left(\gamma \right)}\left(-\Omega_{c}+\nu \left(\sigma_{c}\right)\right)$$

*where $\Omega_{c},\Omega_{u}$ are defined later and a novel robust HOSM control $\nu (\sigma_{c})$ to reject the effect of disturbances is defined as in Equation (24).*

(24) $$\nu \left(\sigma_{c}\right)=-\alpha_{1}\nu_{1}\left(\sigma_{c}\right)-\alpha_{2}\int_{0}^{t}\nu_{2}\left(\sigma_{c}\right)\;dt$$

*with, $\nu_{1}\left(\sigma_{c}\right)={\left|\sigma_{c}\right|}^{\eta_{1}}sign\left(\sigma_{c}\right)$ and $\nu_{2}\left(\sigma_{c}\right)={\left|\sigma_{c}\right|}^{\eta_{2}}sign\left(\sigma_{c}\right)$, $1-2\eta_{1}+\eta_{2}=0$, $1>\eta_{1}\geq 0.5$, and $\alpha_{1},\alpha_{2},\alpha_{3}>0$ are positive constants.*

**Proof.**  The dynamics of the tracking error $\sigma_{c}$ can be expressed as in Equation (25).

(25) $$\begin{array}{cc} {\dot{\sigma }}_{c} & =k_{1}{\dot{e}}_{l}+k_{2}{\ddot{e}}_{l}+k_{3}{\ddot{\delta }}_{d}+k_{4}\lambda_{c}{\dot{x}}_{cf}\\ \; & =\beta f_{1}+{\dot{\psi }}_{v}f_{2}+\Psi_{l}f_{3}+\delta_{d}f_{4}+f_{5}+\Delta_{t}+\left(k_{3}b_{1}-k_{4}\lambda_{c}\right)\mu \left(\gamma \right)T_{fb}\\ \; & =\Omega_{c}+\Omega_{u}\mu \left(\gamma \right)T_{fb}+\Delta_{t} \end{array}$$

where $\Omega_{c}=\beta f_{1}+{\dot{\psi }}_{v}f_{2}+\Psi_{l}f_{3}+\delta_{d}f_{4}+f_{5}$, $\Omega_{u}=k_{3}b_{1}-k_{4}\lambda_{c}$, $f_{1}=k_{1}v_{x}+k_{2}v_{x}a_{11}+2k_{2}l_{s}a_{21}+k_{3}a_{61}$, $f_{2}=2k_{1}l_{s}+k_{2}v_{x}a_{12}+2k_{2}l_{s}a_{22}+k_{2}v_{x}+k_{3}a_{62}$, $f_{3}=k_{1}v_{x}+k_{3}a_{64}$, $f_{4}=k_{2}v_{x}a_{15}+2k_{2}l_{s}a_{25}+k_{3}a_{65}$, $f_{5}=k_{3}a_{63}y_{l}+k_{3}a_{66}{\dot{\delta }}_{d}+k_{4}T_{d}^{s}$, $\Delta_{t}=f_{dt}+f_{dt1}-k_{1}l_{s}v_{x}\rho_{r}+e_{3}\Delta F_{yf}$, $f_{dt}=k_{2}\left[\beta \frac{\partial v_{x}}{\partial dt}+\Psi_{l}\frac{\partial v_{x}}{\partial dt}-l_{s}\frac{\partial v_{x}}{\partial dt}\rho_{r}-l_{s}v_{x}\frac{\partial \rho_{r}}{\partial dt}\right]$, and $f_{dt1}=k_{2}v_{x}\left(e_{1}F_{w}+e_{1}\Delta F_{yf}+e_{4}\Delta F_{yr}\right)+2k_{2}l_{s}\left(e_{2}F_{w}+e_{2}\Delta F_{yf}+e_{5}\Delta F_{yr}\right)-k_{2}\rho_{r}v_{x}^{2}$.

Substituting for the feedback control designed in Equation (23), the error dynamics can be now expressed as in Equation (26).

(26) $${\dot{\sigma }}_{c}=\nu \left(\sigma_{c}\right)+\Delta$$

The lumped disturbance Δ consists of the effects of road curvature, crosswinds, and uncertain tire friction forces. For all practical operating conditions, these disturbances and their time derivatives can be assumed to be bounded. It can be further shown that the lumped disturbance can be divided as $\Delta =\Delta_{1}\left(\sigma_{c}\right)+\Delta_{2}$ with simplifications of the expression in Equation (25). The disturbance terms can be shown to be bounded as in Equation (28).

(27) $$\begin{array}{cc} ∥\Delta_{1}∥ & \leq \chi_{1}∥\sigma_{c}∥ \end{array}$$

(28) $$\begin{array}{cc} |{\dot{\Delta }}_{2}| & \leq \chi_{2} \end{array}$$

where $\chi_{1},\chi_{2}>0$ are any positive parameters.

Now consider the following Lyapunov function in Equation (29).

(29) $$V_{c}=\Sigma^{T}Q_{c}\Sigma$$

with $\Sigma ={\left[\begin{array}{ccc} \nu_{1} & \sigma_{c} & \int_{0}^{t}\nu_{2}\left(\sigma_{c}\right)\;dt \end{array}\right]}^{T}$ and the matrix $Q_{c}=Q_{c}^{T}>0$ denoting a positive definite matrix defined, as [35], in Equation (30).

(30) $$Q_{c}=\frac{1}{2}\left[\begin{array}{ccc} \left(4\alpha_{2}+\alpha_{1}^{2}\right) & \alpha_{1}\alpha_{3} & -\alpha_{1}\\ \alpha_{1}\alpha_{3} & \left(1+\alpha_{3}^{2}\right) & -\alpha_{3}\\ -\alpha_{1} & -\alpha_{3} & 2 \end{array}\right]$$

The above Lyapunov function satisfies the condition in Equation (31).

(31) $$\lambda_{min}{∥\Sigma ∥}^{2}\leq V_{c}\leq \lambda_{max}{∥\Sigma ∥}^{2}$$

with $\lambda_{min},\lambda_{max}$ representing the minimum singular value and the maximum eigenvalue respectively. The rate of evolution of this Lyapunov function can be computed, similarly to [35], as in Equation (32).

(32) $${\dot{V}}_{c}=-\frac{1}{∥\sigma_{c}{∥}^{\left(1-n_{1}\right)}}\Sigma^{T}Q_{c1}\Sigma -\Sigma^{T}Q_{c2}\Sigma$$

where $Q_{c1}$ and $Q_{c2}$ are two positive definite matrices. By the choice of the gains as $\alpha_{1}>{\left[\left(2\alpha_{2}\chi_{1}+\alpha_{3}\chi_{2}-\alpha_{2}\alpha_{3}\right)/\left(2\alpha_{3}-0.5\chi_{1}\right)\right]}^{0.5}$, $\alpha_{2}>\left(2\chi_{2}-\alpha_{1}^{2}\right)/2$, and $\alpha_{3}>\chi_{1}\left(\alpha_{1}^{2}/2+2\alpha_{2}\right)/\left(\alpha_{2}+2\alpha_{1}^{2}-\chi_{2}\right)$, it can be shown, similar to [35], that Equation (33) is valid.

(33) $${\dot{V}}_{c}=-\frac{1}{∥\sigma_{c}{∥}^{\left(1-n_{1}\right)}}\lambda_{min}Q_{c1}{∥\Sigma ∥}^{2}-\lambda_{min}Q_{c2}{∥\Sigma ∥}^{2}$$

Thus, with the proper selection of the gains $\alpha_{i}>0$, the Lyapunov function ${\dot{V}}_{c}$ is negative definite and the sliding surface converges to attain practical bounded stability. □

In the designed closed loop shared control in Theorem 1, the sharing parameter μ(γ) is directly accounted for in the design of the feedback input $T_{fb}$ as shown in Equation (23). Thus, the stability of the DiL closed-loop system in Equation (18) in the presence of road disturbances and tire-friction uncertainties for any authority transfer or shared driving between the driver and the automation system can be ensured.

**Remark 1.**  *In the designed feedback control $T_{fb}$, singularity condition can arise when $\Omega_{u}\mu \left(\gamma \right)\to 0$, i.e., if $(k_{3}b_{1}-k_{4}\lambda_{c})\to 0$ or if μ(γ)→0. However, the modulation factor is a positive bounded entity i.e., $\mu \left(\gamma \right)\in \left[\mu_{min},1\right]$ as presented earlier, and will not result in a singularity condition for the controller. Further, by the selection of the gains κ, $\lambda_{c}$ such that $k_{3}b_{1}\neq k_{4}\lambda_{c}$, the design of the control input would always be feasible.*

**Figure 3:**
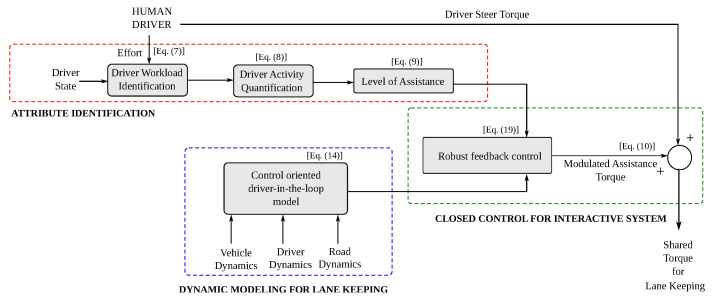
Flow chart of the methodology.

A flowchart for the methodology of implementation of the proposed control scheme is presented in Figure 3.

## 4. Validation and Results

The proposed driver activity adapted cooperative LKA controller was validated on a MATLAB-SIMULINK platform and the SHERPA vehicle simulator for real-time testing.

### 4.1. Simulation Studies

The performance of the proposed approach was evaluated to satisfy the control objectives under the following constraints for safe vehicle operation in Equation (34).

(34) $$|\dot{\psi }|\leq {\dot{\psi }}_{max},|\Psi_{l}|\leq \Psi_{l_{max}},|y_{l}|\leq y_{l_{max}},|\delta_{f}|\leq \delta_{f_{max}},|{\dot{\delta }}_{f}|\leq {\dot{\delta }}_{f_{max}},\left|T_{a}\right|\leq T_{a_{max}}$$

where ${\dot{\psi }}_{max}$ = 0.55 rad/s, $\Psi_{l_{max}}$ = 0.1 rad, $y_{l_{max}}$ = 1.5 m, $\delta_{f_{max}}$ = 0.2 rad, ${\dot{\delta }}_{f_{max}}$ = 0.15 rad/s and $T_{a_{max}}$ = 20 Nm.

For performance evaluations the following controllers were compared:

- **Auto-HOSM**: Autonomous controller (i.e., $T_{d}=0$) with proposed HOSM control law.
- **CLKA-HOSM**: Shared controller with proposed HOSM control law.

The sliding surface gains, defined in Equation (22), without the sharing parameter term were obtained using particle swarm optimization (PSO) [46] for optimal results. Accordingly, each particle was defined as $X=\left[\begin{array}{ccc} k_{1} & k_{2} & k_{3} \end{array}\right]$. Consequently, the particles were able to obtain the optimal solutions for the gains based on an objective function which was formulated to minimize the lane tracking errors and satisfy the system constraints in Equation (34) discussed earlier. We considered particle size as 20 and a total of 100 iterations for the PSO algorithm. Using the PSO approach, the sliding surface gains were computed as $k_{1}=3.6085$, $k_{2}=10.5804$, and $k_{3}=0.9706$. Subsequently, the gains of the novel STA controller were selected as $\alpha_{1}$ = 33.9379, $\alpha_{2}=150$, $\alpha_{3}=11.2697$ and β=0.6383 for normal road conditions with unity road friction. The conflict parameter gains were chosen as $k_{4}=0.001$, $\lambda_{c}=1.5$, respectively.

To replicate the human driver torque for the simulation study, a dynamic model based on neuromuscular attributes, time-lags, etc., as discussed in [9,43] was employed. Employing this driver model with varying parameters, the virtual driver torque for simulations was replicated. For all validation purposes, the driver gains were considered as $K_{c}=8.57$ and $K_{a}=15.75$ respectively. Accounting for the mental workload, two driver states i.e., watchful and distracted to compute the driver state variable DS were considered. During the distracted mode, the external driver torque input was scaled by a factor of 0.2 to represent a distracted driver.

**Figure 4:**
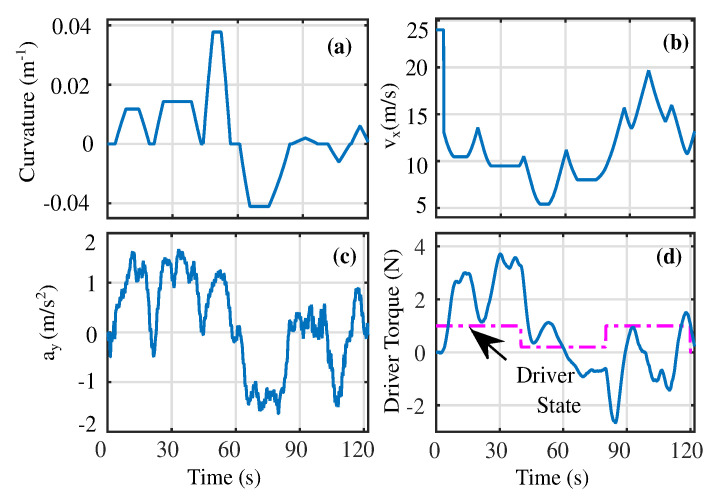
The road and driver input conditions: (**a**) Curvature; (**b**) Longitudinal Velocity; (**c**) Lateral Acceleration; (**d**) Driver input torque.

The simulations were performed on the Satory test track [5] as shown in Figure 4a under variable longitudinal velocity conditions i.e., $v_{x}\in \left[5,25\right]$ m/s shown in Figure 4b. The lateral acceleration of the vehicle is limited to $|a_{y}{|}_{max}\leq 2\;m/s^{2}$, indicating normal driving conditions as shown in Figure 4c. To evaluate the shared control performance, we considered the human driver to be distracted between t∈[40,80] s while during the rest of the driving cycle, the driver was watchful. Accordingly, the input driver torque reflecting such conditions is shown in Figure 4d.

**Figure 5:**
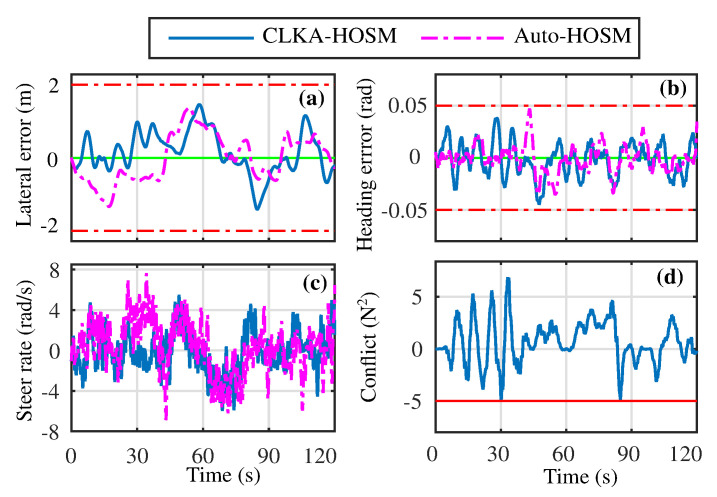
The lane tracking and driver comfort performance for the proposed controller (CLKA-HOSM) and the autonomous controller (Autonomous-HOSM). (**a**) Lateral deviation error; (**b**) Heading error; (**c**) steering rate; (**d**) Conflict product of driver and automation torques.

The performance of the Auto-HOSM and CLKA-HOSM controllers are presented in Figure 5a–d along with that of the Auto-HOSM. Both controllers ensured that the lane tracking errors and the steering rate were within the prescribed limits discussed earlier. As the shared controller incorporates human action in the control process, the above performance indicators of the shared controller have a higher magnitude that their autonomous counterpart. The root mean square (rms) and maximum values of the above indicators for the Auto-HOSM controller were computed as $y_{l_{rms}}=0.57$, $\Psi_{l_{rms}}=0.0131$, ${\dot{\delta }}_{d_{rms}}=2.5917$ and $|y_{l}{|}_{max}=1.19$, $|\Psi_{l}{|}_{max}=0.0469$, $|{\dot{\delta }}_{d}{|}_{max}=7.6202$, respectively. Similarly, the performance metrics of the CLKA-HOSM controller were $y_{l_{rms}}=0.5267$, $\Psi_{l_{rms}}=0.0162$, ${\dot{\delta }}_{d_{rms}}=2.0609$, and $|y_{l}{|}_{max}=1.2750$, $|\Psi_{l}{|}_{max}=0.0446$, $|{\dot{\delta }}_{d}{|}_{max}=5.9638$. Such performance metrics indicate good lane-keeping performance for both controllers. Further, the steering rate performance shows improvement under the proposed CLKA-HOSM controller.

Along with such lane-keeping performance, the conflict between the human driver and the autonomous controller for the CLKA-HOSM controller is also presented in Figure 5d. Using the proposed controller, the conflict is kept within limits such that, $T_{d}T_{a}>-5\;N^{2}$.

**Figure 6:**
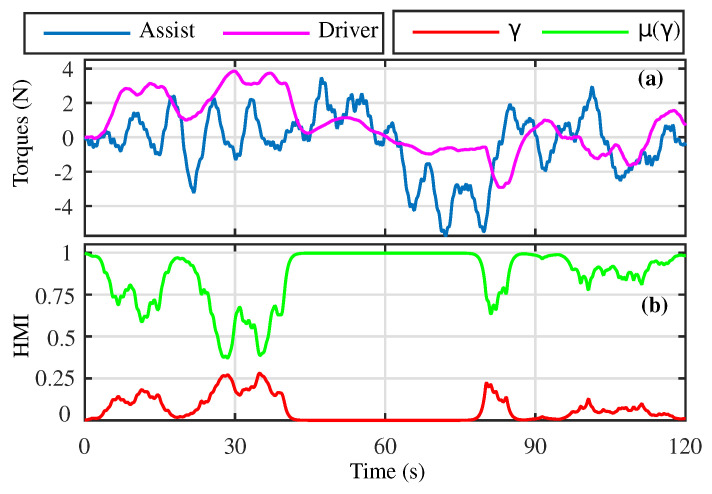
The HMI under the CLKA-HOSM controller. (**a**) Driver and Assistance Torque; (**b**) Driver activity and the level of assistance provided.

For further illustration of the shared control performance, the torques generated by the human driver and autonomous agent along with the driver activity–performance indicators are presented in Figure 6. Based on the driver’s activity, the level of assistance torque generated varies for completing the driving task.

To assess the performance of shared control activity, the following metrics [42] were also a time interval η:

(35) $$\begin{array}{c} \mathrm{AFac}=\frac{T_{d_{pow}}}{T_{a_{pow}}},\;\;\mathrm{SW}=\frac{1}{\eta }\int_{0}^{\eta }T_{a}\left(t\right)T_{d}\left(t\right){\dot{\delta }}_{d}\left(t\right)dt,\;\; \end{array}$$

- AFac: Denotes the ratio between efforts generated by the automation and human driver for completing the driving task i.e., in Equation (36).
  (36) $$\mathrm{AFac}=\frac{T_{d_{pow}}}{T_{a_{pow}}}$$
  If the values of AFac >1, the assistance provided by the automation is less than that of the driver, and inversely for AFac <1.
- SW: This indicates the steering workload and is representative of the effort generated by both agents simultaneously for completing the driving task i.e., in Equation (37).
  (37) $$\mathrm{SW}=\frac{1}{\eta }\int_{0}^{\eta }T_{a}\left(t\right)T_{d}\left(t\right){\dot{\delta }}_{d}\left(t\right)dt$$
  A larger magnitude of negative steering workload indicates that the assistance provided by the automation to the human driver is not good for shared control.

For efficient shared control, the AFac should be less than 1 and the negative steering workload should be low. Using the proposed CLKA-HOSM controller, these metrics are computed as AFac = 0.8192 and Negative SW = −206.6476, indicating a good quality of shared control. To assess the shared control performance further, performance analysis was performed for a shared controller based on the proposed HOSM control law, but with no conflict parameter i.e., $k_{4}=0$ and $\lambda_{c}=0$ (**SC-NoK4**) and is presented in Table 1. Please note that the values of Neg SW (i.e., negative steer workload) and ${T_{d}T_{a}}_{min}$ (i.e., maximum value of conflict) is less than zero.

With the increase in the magnitude of $k_{4}$, the negative steer workload and the maximum values of conflict increase showing deteriorating shared control performance. Further, the lateral error also increases, from a minimum of 1.219 m to a maximum of 1.889 m, as more control is passed on to the human driver, from $K_{4}=0$ to $K_{4}=0.01$. Similar performance is seen with the increase in values of $\lambda_{c}$ as well, from $\lambda_{c}=0$ to $\lambda_{c}=2$. From the presented results, the best performance in terms of lane errors, $|y_{l}{|}_{max}=1.624$, and conflict reduction, $T_{d}T_{a}=5.242$, is obtained for $k_{4}=0.001$ and $\lambda_{c}=0.5$. Further, the presence of the gains $k_{4}$ and $\lambda_{c}$ improves the performance of the controller, in terms of conflict minimization and negative SW, in comparison to the case when $k_{4}=0$ and $\lambda_{c}=0$ across all aspects.

To ascertain the robustness of the proposed CLKA-HOSM controller, random parametric uncertainties in the vehicle and driver parameters were considered. Specifically, uncertain values of *M*, $I_{z}$, and $I_{s}$ which are susceptible to the payload, wear, tear, etc. are employed. Similarly, the uncertainty in driver model parameters $K_{a}$ and $K_{c}$ to account for various driver behaviors are also considered. The lane-keeping and conflict reduction performance of the CLKA-HOSM (i.e., C1) and SC-NoK4 (i.e., C2) controllers under influence of such uncertainties are presented in Table 2.

Under the influence of vehicle and driver uncertainties up to 20%, the proposed CLKA-HOSM controller performs well in ensuring lane keeping ($|y_{l}|{}_{rms}$ = 0.52, $|\Psi_{l}|{}_{rms}$ = 0.017 for CLKA-HOSM, against $|y_{l}|{}_{rms}$ = 0.47, $|\Psi_{l}|{}_{rms}$ = 0.018 for SC-NoK4) and also minimizing the conflict between driver and autonomous system (${T_{d}T_{a}}_{min}$ = 6.705 for CLKA-HOSM, against ${T_{d}T_{a}}_{min}$ = 7.169 for SC-NoK4). The CLKA-HOSM controller outperforms the SC-NoK4 controller in handling uncertainties, and thus establishes the significance of the gains $k_{4}$ and $\lambda_{c}$ in performance enhancement.

### 4.2. Experimental Results: SHERPA Vehicle Simulator

**Figure 7:**
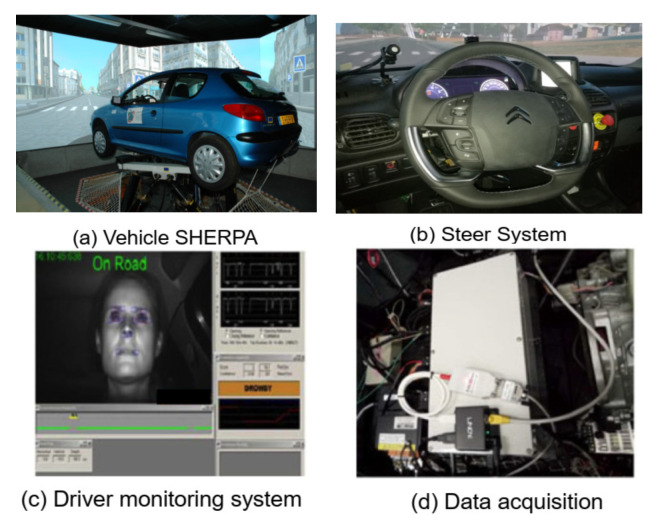
Experimental setup for the SHEPRA vehicle simulator.

The shared DiL-LKA approach was validated in real-time on the SHERPA vehicle simulator shown in Figure 7.

The SHERPA simulator is built using a modified Peugot 206 vehicle on a Stewart platform and is composed of multiple modules for handling driving-related tasks such as perception, path planning, driver monitoring, and human–machine interface management. For more details on the SHERPA simulator, refer to [5]. Using the driving monitoring unit, the driver state is directly available as a binary input while the torque is measured via a sensor on the steering wheel. With haptic feedback via the steering wheel provided, this simulator setup has been used for validation of direct shared control works [5,43] similar to that proposed in this work.

**Figure 8:**
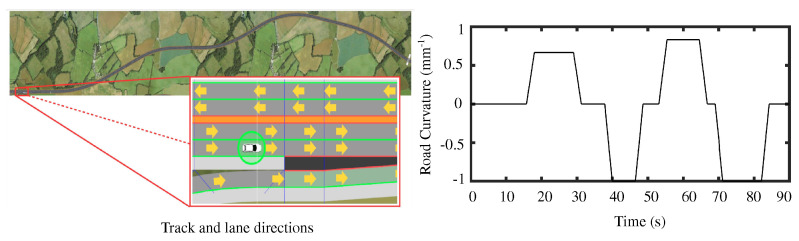
CoCoVeA track and lanes directions along with road curvature of the sections.

Using the SHERPA setup (with a discretization time of 0.01 s), we now present illustrative results to highlight the lane-keeping and conflict-reduction performance of the proposed shared DiL controller in this work to further support our earlier presented simulation-based analysis. All performance evaluations on the SHERPA simulator are made on a test track represented in Figure 8 that comes from the CoCoVeA project (Cooperation Conductor-Véhicule Automatisé).

The results for the Auto-HOSM controller robustness against longitudinal speed and the friction variations are first presented to highlight the robustness of the proposed novel control law. For multiple driving tests performed, the aggregated results are presented in Table 3. It can be seen that the variations in the longitudinal speed of the vehicle and the road friction do not affect the performance of the proposed controller. The controller ensures good trajectory tracking by maintaining the lateral deviation below $|y_{l}|{}_{max}$ = 0.5824 < 1.5 m, the maximum heading error below $|\Psi_{l}|{}_{max}$ = 0.0074 < 0.1 rad, without saturating the motor control of the steering system $|T_{a}|{}_{max}$ = 1.2104 < 20 N·m.

**Figure 9:**
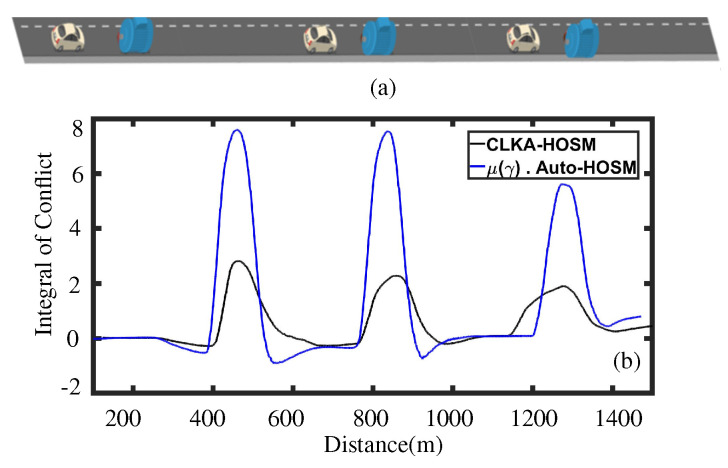
(**a**) The obstacle avoidance scenario; (**b**) comparison between the LKA controller weighted with the LOA function and the CLKA-HOSM controller for the minimization of the conflict for an obstacle avoidance scenario using a metric Integral of Conflict.

In the second case, the proposed CLKA-HOSM controller for an obstacle avoidance scenario is tested with sharing parameter values as $k_{4}$ = 15 and $\lambda_{c}$ = 0.5. Accordingly, as shown in Figure 9, three obstacles were placed on the road, and the driver was asked to avoid them by changing the lane. For comparisons, the same test was also repeated with the Auto-HOSM controller weighted by the LOA function presented in Equation (12). The performance results for both controllers are presented in Figure 9.

In Figure 9b, the metric *Integral of Conflict* is defined as $IOC=-\frac{1}{\tau }\int_{0}^{T}T_{a}\left(t\right)T_{d}\left(t\right)dt$ for a time period τ. It was observed that the CLKA-HOSM controller is more efficient in terms of conflict minimization i.e., the maximum value of the integral of the conflict is 2.7. On the other hand, the maximum value of the integral of the conflict for the Auto-HOSM controller weighted with the LOA function was 7.8. In the case of the proposed CLKA-HOSM controller, AFac = 0.8818, Negative SW = 14.9646, and ${\left(T_{d}T_{a}\right)}_{min}$ = −4.8727 was obtained. In contrast, for the Auto-HOSM controller weighted with the LOA function, AFac = 0.974, Negative SW = 108.218, and ${\left(T_{d}T_{a}\right)}_{min}$ = −24.539 were obtained. Such results show that the proposed CLKA-HOSM outperforms the other design in terms of shared control performance.

For further analysis of the shared control performance, the parameters $k_{4}$ and $\lambda_{c}$ were varied and tests were performed. Performance results for the CLKA-HOSM controller under such variations are shown in Table 4.

It can be seen in Table 4 that the shared parameters have a significant impact on the AFac metric, from 0.4794 to 1.0604, and SW metric, from −14.9646 to −348.7971. The chosen best combination values of these metrics using the proposed CLKA-HOSM controller are AFac = 0.8818 and SW = −14.9646, indicating a good quality of shared control. From the presented results, the best performance in terms of conflict reduction is obtained for the combination $k_{4}=15$ and $\lambda_{c}=0.5$.

## 5. Conclusions

In this work, a novel robust shared controller for a DiL-lane-keeping assistance system was proposed and evaluated. The HMI was managed via an adaptive mapping which reflected driver performance corresponding to the identified physical and mental workload of the driver. Along with lane tracking errors and driver comfort enhancement, the issue of conflict between the driver and autonomous controller was also addressed by the introduction of a novel sharing parameter. Addressing such objectives, a novel higher-order sliding mode control algorithm was proposed and its stability for the closed-loop DiL system affected by disturbances was established.

The performance of the proposed controller was evaluated via simulations and experiments on the SHERPA vehicle simulator for different longitudinal velocity, different road friction conditions, time-varying road curvatures of the Satory test track, parametric uncertainties, and for obstacle avoidance scenarios. Comparison between the fully autonomous controller, the proposed sharing control law without the introduction of the novel parameter for conflict reduction, and the proposed sharing control law with the introduction of this minimization parameter was extensively discussed. From the experimental results, it can be seen that the fully autonomous controller achieved the best lane tracking and heading error performances (30% better than the sharing control law), but the sharing control law achieved the best conflict minimization (65.38% better than the sharing control law without the introduction of this novel term). Further, the cooperative driving quality improved by 9.4%, and the negative steering workload was reduced by 86.13% in comparison to the Auto-HOSM controller showing the efficiency of the proposed controller.

The proposed controller was constructed in order to deal with the goals of lane maintenance, driver comfort improvement, and conflict reduction, which fill a particular need in improving the driving experience for road vehicle transportation. In the future, the driver activity function will be enhanced by including the driving style, skill, and other attributes reflecting a wider variety of driver behaviors. An expansion of the proposed cooperative architecture to the cruise and integrated longitudinal–lateral control will be carried out.

## Figures and Tables

**Table 1:** Influence of $k_{4}$ and $\lambda_{c}$ on HMI.

Case	$\lambda_{c}$	$|y_{l}|{}_{\max}$ (m)	AFac	Neg. SW (N${}^{2}$m${}^{2}$rad/s)	${T_{d}T_{a}}_{\min}$ (N${}^{2}$m${}^{2}$)
$k_{4}=0$	0	1.219	0.735	219.3	9.132
$k_{4}=0.001$	0.5	1.624	0.843	202.1	5.242
	1.5	1.275	0.819	206.6	5.22
	2	1.389	0.818	209.6	5.92
$k_{4}=0.01$	0.5	1.241	0.824	216.7	8.35
	1.5	1.889	0.763	223.2	6.577
	2	1.228	0.723	219.6	8.308

**Table 2:** Influence of uncertainties on controller performance.

Unct.	Cont.	$|y_{l}|{}_{\mathrm{rms}}$	$|\Psi_{l}|{}_{\mathrm{rms}}$	$|{\dot{\delta }}_{d}|{}_{\mathrm{rms}}$	${T_{d}T_{a}}_{\min}$	Neg. SW	AF
5%	C1	0.508	0.019	2.054	6.514	226.4	0.797
	C2	0.659	0.022	2.375	11.624	235.2	0.811
15%	C1	0.554	0.016	2.378	8.352	211.5	0.722
	C2	0.585	0.016	2.252	7.599	206.8	1.01
20%	C1	0.5221	0.0172	2.165	6.705	204.5	0.822
	C2	0.472	0.0182	2.037	7.169	208.2	0.599

**Table 3:** Influence of speed variation and friction on the performance of the lane-keeping controller.

v${}_{x}$ (m/s)	Friction	$|y_{l}|{}_{\max}$ (m)	$|\Psi_{l}|{}_{\max}$ (rad)	$|T_{a}|{}_{\max}$ (N·m)
14	1	0.1116	0.0024	0.2523
	0.6	0.1188	0.0063	0.2679
	0.4	0.1289	0.0024	0.2557
20	1	0.3213	0.0045	0.6807
	0.6	0.3211	0.0044	0.6783
	0.4	0.3111	0.0044	0.6663
25	1	0.5727	0.0074	1.2104
	0.6	0.5824	0.0072	1.1257
	0.4	0.5792	0.0073	1.1432

**Table 4:** Influence of $k_{4}$ and $\lambda_{c}$ on HMI.

Case	$\lambda_{c}$	AFac	Neg. SW (N${}^{2}$m${}^{2}$rad/s)	${T_{d}T_{a}}_{\min}$ (N${}^{2}$m${}^{2}$)
$k_{4}=-5$	0.5	0.5923	348.7971	−61.0736
	0.8	0.4794	118.1967	−70.3217
$k_{4}=-1$	0.5	0.9754	91.0878	−35.5465
	0.8	0.9507	138.8792	−36.7558
$k_{4}=0$	0	1.0192	106.8268	−27.2865
$k_{4}=5$	0.5	1.0240	108.8057	−18.0648
	0.8	1.0604	108.0537	−15.0333
$k_{4}=10$	0.5	0.9909	40.4668	−7.5843
	0.8	0.9515	36.0443	−8.4723
$k_{4}=15$	0.5	0.8818	14.9646	−4.8727
	0.8	0.9499	18.4843	−7.2032

## Data Availability

Not applicable.

## Nomenclature

Symbol	Description	Value
*M*	total mass of the vehicle	2025 [kg]
$l_{f}$	distance from CoG to front axle	1.3 [m]
$l_{r}$	distance from CoG to rear axle	1.6 [m]
$t_{p}$	tire length contact	0.052 [m]
$l_{s}$	look-ahead distance	5 [m]
$I_{z}$	vehicle yaw moment of inertia	2800 [kgm${}^{2}$]
$I_{s}$	steering moment of inertia	0.05 [kgm${}^{2}$]
$R_{s}$	steering gear ratio	16.3 [-]
$B_{u}$	steering system damping	2.5 [N/rad]
$C_{pf}$	front cornering stiffness	42,500 [N/rad]
$C_{pr}$	rear cornering stiffness	57,000 [N/rad]
$R_{s}$	rate driver’s—vehicle’s wheel angles	
$w_{r}$	width of the vehicle	
β	side slip angle	
δ	steering angle	
$\delta_{d}$	steering angle	
${\dot{\delta }}_{d}$	steering rate	
ψ	heading angle	
$F_{yf}$	front friction force	
$F_{yr}$	rear friction force	
$v_{x}$	longitudinal velocity	
$\alpha_{f}$	front slip angle	
$\alpha_{r}$	rear slip angle	
$T_{s}$	self-aligning torque	
$\Delta F_{i}$	uncertainty of tire friction force	
$K_{p}$	level of assistance	
$y_{l}$	lateral deviation error	
$\Psi_{l}$	orientation error w.r.t the lane center-line	
$T_{d}$	driver torque	
$T_{a}$	automation assistance torque	
μ(γ)	rate driver workload-based performance—LOA	
$T_{fb}$	feedback control torque	
$K_{a}$	anticipatory gain	
$K_{c}$	compensatory gain	
$\theta_{near}$	near visual points of the driver	
$\theta_{far}$	far visual points of the driver	
$\lambda_{c}$	the level of sharing	
$T_{d}^{s}$	driver torque measured at the steering wheel	
${\dot{x}}_{cf}$	conflict dynamics	
$\sigma_{c}$	linear error surface of the SMC	
AFac	ratio between automation and human	
**Acronyms**		
Symbol	Description	
ADAS	Advanced driver assist system	
LKA	Lane keeping assistance	
ACC	Adaptive cruise control	
CA	Collision avoidance	
HMI	Human machine interaction	
DiL	Driver-in-the-loop	
HOSM	High order sliding mode	
SMC	Sliding mode control	
BT	Brush-Tire	
DS	Driver state	
DMU	Driver monitoring unit	
LOA	Level of assistance	
Auto-HOSM	Autonomous controller with proposed HOSM control	
CLKA-HOSM	Shared controller with proposed HOSM control	
SC-NoK4	Shared controller with proposed HOSM control with $K_{4}=0$	
PSO	Particle swarm optimization	
SW	Steering workload	
IOC	Integral of conflict	
SHERPA	Simulateur Hybride d’Etude et de Recherche Pour l’Automobile	

## References

[1] Rajamani R. (2012). Vehicle Dynamics and Control.

[2] Flemisch F., Kelsch J., Loper C., Schieben A., Schindler J., Heesen M. Cooperative control and active interfaces for vehicle assistance and automation. Proceedings of the FISITA World Automotive Congress.

[3] Abbink D.A., Mulder M., Boer E.R. (2011). Haptic shared control: Smoothly shifting control authority?. Cogn. Technol. Work.

[4] Saito T., Wada T., Sonoda K. (2018). Control Authority Transfer Method for Automated-to-Manual Driving Via a Shared Authority Mode. IEEE Trans. Intell. Veh..

[5] Nguyen A.T., Sentouh C., Popieul J.C. (2017). Driver-automation cooperative approach for shared steering control under multiple system constraints: Design and experiments. IEEE Trans. Ind. Electron..

[6] Wada T., Sonoda K., Tada S. (2016). Simultaneous Achievement of Supporting Human Drivers and Improving Driving Skills by Shared and Cooperative Control. IFAC-PapersOnLine.

[7] Saleh L., Chevrel P., Claveau F., Lafay J.F., Mars F. (2013). Shared steering control between a driver and an automation: Stability in the presence of driver behavior uncertainty. IEEE Trans. Intell. Transp. Syst..

[8] Schnelle S., Wang J., Su H., Jagacinski R. (2017). A Driver Steering Model with Personalized Desired Path Generation. IEEE Trans. Syst. Man, Cybern. Syst..

[9] Sentouh C., Chevrel P., Mars F., Claveau F. A sensorimotor driver model for steering control. Proceedings of the IEEE International Conference on Systems, Man and Cybernetics.

[10] Li L., Liu Y., Wang J., Deng W., Oh H. (2016). Human dynamics based driver model for autonomous car. IET Intell. Transp. Syst..

[11] Tanaka Y., Kashiba Y., Yamada N., Suetomi T., Nishikawa K., Nouzawa T., Tsuji T. Active-steering control system based on human hand impedance properties. Proceedings of the 2010 IEEE International Conference on Systems, Man and Cybernetics.

[12] Sharma O., Sahoo N.C., Puhan N.B. (2021). Recent advances in motion and behavior planning techniques for software architecture of autonomous vehicles: A state-of-the-art survey. Eng. Appl. Artif. Intell..

[13] Gambhire S.J., Kishore D.R., Londhe P.S., Pawar S.N. (2021). Review of sliding mode based control techniques for control system applications. Int. J. Dyn. Control.

[14] Wu J., Zhang J., Tian Y., Li L. (2021). A Novel Adaptive Steering Torque Control Approach for Human–Machine Cooperation Autonomous Vehicles. IEEE Trans. Transp. Electrif..

[15] Kumar V., Naresh R., Sharma V., Kumar V. (2022). State-of-the-Art Optimization and Metaheuristic Algorithms. Handbook of Intelligent Computing and Optimization for Sustainable Development.

[16] Brizuela-Mendoza J.A., Sorcia-Vázquez F.D.J., Rumbo-Morales J.Y., Lozoya-Ponce R.E., Rodríguez-Cerda J.C. (2021). Active fault tolerant control based on eigenstructure assignment applied to a 3-DOF helicopter. Asian J. Control.

[17] Rumbo Morales J.Y., Brizuela Mendoza J.A., Ortiz Torres G., Sorcia Vázquez F.d.J., Rojas A.C., Pérez Vidal A.F. (2022). Fault-Tolerant Control implemented to Hammerstein–Wiener model: Application to Bio-ethanol dehydration. Fuel.

[18] Xing Y., Lv C., Cao D., Hang P. (2021). Toward human-vehicle collaboration: Review and perspectives on human-centered collaborative automated driving. Transp. Res. Part C Emerg. Technol..

[19] Wang J., Zhang G., Wang R., Schnelle S.C., Wang J. (2017). A Gain-Scheduling Driver Assistance Trajectory-Following Algorithm Considering Different Driver Steering Characteristics. IEEE Trans. Intell. Transp. Syst..

[20] Soualmi B., Sentouh C., Popieul J., Debernard S. (2014). Automation-driver cooperative driving in presence of undetected obstacles. Control Eng. Pract..

[21] Wang Z., Zheng R., Nacpil E.J.C., Nakano K. (2022). Modeling and analysis of driver behaviour under shared control through weighted visual and haptic guidance. IET Intell. Transp. Syst..

[22] Mars F., Deroo M., Hoc J.M. (2014). Analysis of Human-Machine Cooperation When Driving with Different Degrees of Haptic Shared Control. IEEE Trans. Haptics.

[23] Boink R., van Paassen M.M., Mulder M., Abbink D.A. Understanding and reducing conflicts between driver and haptic shared control. Proceedings of the 2014 IEEE International Conference on Systems, Man, and Cybernetics (SMC).

[24] Huang C., Lv C., Hang P., Hu Z., Xing Y. (2022). Human–Machine Adaptive Shared Control for Safe Driving Under Automation Degradation. IEEE Intell. Transp. Syst. Mag..

[25] Li X., Wang Y., Su C., Gong X., Huang J., Yang D. (2022). Adaptive Authority Allocation Approach for Shared Steering Control System. IEEE Trans. Intell. Transp. Syst..

[26] Deng H., Zhao Y., Feng S., Wang Q., Lin F. (2022). Shared Control for Intelligent Vehicle Based on Handling Inverse Dynamics and Driving Intention. IEEE Trans. Veh. Technol..

[27] Flemisch F., Nashashibi F., Rauch N., Schieben A., Glaser S., Temme G., Resende P., Vanholme B., Löper C., Thomaidis G. Towards highly automated driving: Intermediate report on the HAVEit-joint system. Proceedings of the 3rd European Road Transport Research Arena.

[28] Sentouh C., Nguyen A.T., Benloucif M.A., Popieul J.C. (2019). Driver-Automation Cooperation Oriented Approach for Shared Control of Lane Keeping Assist Systems. IEEE Trans. Control Syst. Technol..

[29] Shimizu Y., Kawai T., Yuzuriha J. (1999). Improvement in Driver-Vehicle System Performance by Varying Steering Gain with Vehicle Speed and Steering Angle: VGS (Variable Gear-Ratio Steering System).

[30] Izadi V., Ghasemi A.H. (2021). Modulation of control authority in adaptive haptic shared control paradigms. Mechatronics.

[31] Nguyen A.T., Sentouh C., Popieul J.C., Soualmi B. Shared Lateral Control with Online Adaptation of the Automation Degree for Driver Steering Assist System: A Weighting Design Approach. Proceedings of the IEEE 54th Annual Conference on Decision and Control (CDC).

[32] Oufroukh N.A., Mammar S. Integrated driver co-pilote approach for vehicle lateral control. Proceedings of the 2014 IEEE Intelligent Vehicles Symposium Proceedings.

[33] Lv C., Wang H., Cao D., Zhao Y., Sullman M., Auger D.J., Brighton J., Matthias R., Skrypchuk L., Mouzakitis A. A Novel Control Framework of Haptic Take-Over System for Automated Vehicles. Proceedings of the 2018 IEEE Intelligent Vehicles Symposium (IV).

[34] Ahn C., Peng H., Tseng H.E. Robust estimation of road friction coefficient. Proceedings of the 2011 American Control Conference.

[35] Rath J.J., Veluvolu K.C., Defoort M., Soh Y.C. (2014). Higher-order sliding mode observer for estimation of tyre friction in ground vehicles. IET Control Theory Appl..

[36] Moreno J.A., Osorio M. (2012). Strict Lyapunov Functions for the Super-Twisting Algorithm. IEEE Trans. Autom. Control.

[37] Ahn C., Peng H., Tseng H.E. (2013). Robust Estimation of Road Frictional Coefficient. IEEE Trans. Control Syst. Technol..

[38] Kiencke U., Nielsen L. (2005). Automotive Control Systems.

[39] Baffet G., Charara A., Lechner D., Thomas D. (2008). Experimental evaluation of observers for tire–road forces, sideslip angle and wheel cornering stiffness. Veh. Syst. Dyn..

[40] Nguyen A.T., Sentouh C., Popieul J.C. Online Adaptation of the Authority Level for Shared Lateral Control of Driver Steering Assist System Using Dynamic Output Feedback Controller. Proceedings of the 41st Annual Conference of the IEEE Industrial Electronics Society.

[41] Nguyen A.T., Rath J.J., Lv C., Guerra T.M., Lauber J. (2021). Human-Machine Shared Driving Control for Semi-Autonomous Vehicles Using Level of Cooperativeness. Sensors.

[42] Rath J.J., Senouth C., Popieul J.C. (2019). Personalised lane keeping assist strategy: Adaptation to driving style. IET Control Theory Appl..

[43] Bencloucif M., Nguyen A.T., Sentouh C., Popieul J. (2019). Cooperative Trajectory Planning for Haptic Shared Control between Driver and Automation in Highway Driving. IEEE Trans. Indus. Electron..

[44] Dong J., Yang G.H. (2011). Control synthesis of T-S fuzzy systems based on a new control scheme. IEEE Trans. Fuzzy Syst..

[45] Reymond G., Kemeny A., Droulez J., Berthoz A. (2001). Role of Lateral Acceleration in Curve Driving: Driver Model and Experiments on a Real Vehicle and a Driving Simulator. Hum. Factors J. Hum. Factors Ergon. Soc..

[46] Poli R., Kennedy J., Blackwell T. (2007). Particle swarm optimization. Swarm Intell..

